# Heat-Induced Changes in κ-Carrageenan-Containing Chocolate-Flavoured Milk Protein Concentrate Suspensions under Controlled Shearing

**DOI:** 10.3390/foods12244404

**Published:** 2023-12-07

**Authors:** Anushka Mediwaththe, Thom Huppertz, Jayani Chandrapala, Todor Vasiljevic

**Affiliations:** 1 Advanced Food Systems Research Unit, Institute of Sustainable Industries & Liveable Cities, College of Sports, Health and Engineering, Victoria University, Werribee Campus, Werribee, VIC 3030, Australia; anushka.mediwaththe@live.vu.edu.au (A.M.); thom.huppertz@frieslandcampina.com (T.H.); 2 FrieslandCampina, 3818 LE Amersfoort, The Netherlands; 3 Food Quality and Design Group, Wageningen University & Research, 6708 WG Wageningen, The Netherlands; 4 School of Science, RMIT University, Bundoora, VIC 3083, Australia; jayani.chandrapala@rmit.edu.au

**Keywords:** milk protein concentrate, heat stability, shear, κ-carrageenan, chocolate flavour

## Abstract

Milk protein dispersions containing added cocoa powder (1.5% (*w*/*w*)) and sucrose (7% (*w*/*w*)) and varying levels of κ-carrageenan (0.01, 0.03, or 0.05% *w*/*w*) were subjected to combined heat treatment (90 °C/5 min or 121 °C/2.6 min) and shear (100 or 1000 s^−1^) to investigate the heat stability of milk proteins. The application of shear led to a notable reduction in non-sedimentable proteins, resulting in an increase in the average particle size and apparent viscosity of the dispersions, particularly at high concentrations of k-carrageenan and elevated temperatures. This indicates that shear forces induced prominent protein aggregation, especially at higher κ-carrageenan concentrations. This aggregation was primarily attributed to the destabilisation of micelles and presence of loosely bound caseins within the κ-carrageenan network, which exhibited increased susceptibility to aggregation as collision frequencies increased due to shear.

## 1. Introduction

In the marketplace, dairy beverages are available in diverse flavours, including chocolate, coffee, strawberry, and vanilla, as well as options with reduced or no fat. Among flavoured milk products, high-protein ready-to-drink (RTD) dairy beverages have gained popularity, designed for meal replacements and sports enthusiasts [1]. To achieve high protein content, milk protein concentrate (MPC) is commonly used, known for its substantial protein content ranging from 50% to 85% per dry solid content [2].

In chocolate-flavoured milk beverages, cocoa powder is often used as a flavouring agent. A significant proportion of cocoa powder particles remains insoluble when mixed with milk protein dispersions. As a result, during storage, some cocoa particles tend to settle at the bottom of the container due to the gravitational force [3]. This can be prevented by using hydrocolloid stabilizers such as κ-carrageenan. Such stabilizers create a gel network with milk protein that traps cocoa particles, reducing sedimentation, fat separation, and protein gelation [4]. 

The most recognised theory on the mechanism of κ-carrageenan’s action in milk, specifically its interaction with casein micelles, involves the κ-carrageenan creating a weak gel, which holds the casein micelles suspended, preventing phase separation and increasing milk viscosity, aiding micellar stability, even below the critical gel concentration [5]. The carrageenan–milk protein interactions are influenced by the interactions among milk proteins themselves [6]. Heat treatment strengthens carrageenan gels in milk by displacing carrageenan complexed with κ-casein (CN) due to denatured β-lactoglobulin (LG). This increases carrageenan availability for carrageenan–carrageenan interactions, enhancing long-term product stability [7]. Nevertheless, during sterilization, challenges such as undesired flocculation, coagulation, and sediment formation may arise due to increased viscosity and flavour–milk interactions [8]. The inappropriate use of stabilizers can lead to the manifestation of these undesirable characteristics in the final product.

Next to heat treatment, flavoured milk beverages with added κ-carrageenan undergo a range of processing stages, including pumping, blending, agitating, homogenizing, and passing through heat exchangers. These processes expose the beverages to hydrodynamic shear forces. These shear forces can induce structural changes of varying degrees to milk proteins [9]. While the combined effects of heating and shearing are commonly encountered in thermal processing, there is limited research on how these combined effects influence structural changes and protein interactions in complex milk systems, particularly in relation to milk protein concentrates (MPCs) during the production of chocolate flavoured high protein dairy beverages with varying levels of κ-carrageenan. Therefore, the objective of this study was to investigate the heat stability of chocolate-flavoured 8% MPC dispersion under controlled shearing at different heating regimes and varying levels of added κ-carrageenan. The processing conditions employed in this study closely resembled those used in commercial settings, such as a temperature of 90 °C for 5 min and 121 °C for 2.6 min and shear rates of 100 and 1000 s^−1^. However, it is worth noting that lower heating rates were employed due to equipment limitations compared to industrial conditions.

## 2. Materials and Methods

### 2.1. Materials

MPC powder was procured from Fonterra Co-operative (Palmerston North, New Zealand) and stored in airtight plastic containers at a temperature of −20 °C. The composition of the MPC powder was 81.0% (*w*/*w*) total proteins, 1.6% (*w*/*w*) fat, 5.5% (*w*/*w*) carbohydrates, and 7.2% (*w*/*w*) ash, according to the manufacturer’s specifications. All the chemicals used for the analysis were obtained from Sigma-Aldrich Pty Ltd. (Castle Hill, NSW, Australia). Ultrapure water (Milli-Q water by Merck Millipore in Bayswater, Vic, Australia) was used throughout the experiments. 

### 2.2. Sample Preparation and Treatment

The MPC powder was reconstituted in Milli-Q water to obtain dispersions containing ~8% protein (*w*/*w*). The solution was continuously stirred for 1 h at 50 °C for the complete solubilisation of the powder [10]. A total of 1.5% cocoa powder (*w*/*w*) and 7% (*w*/*w*) sucrose were added to milk protein dispersions according to the typical composition of chocolate flavoured milk [11]. The κ-carrageenan concentration, selected from the recommended range commonly used in chocolate milk formulations [12], was 0.01%, 0.03%, or 0.05% (*w*/*w*) and was achieved by adding appropriate amount to the MPC dispersions prepared as mentioned above. The pH of all samples was adjusted to 6.8 via the slow addition of disodium phosphate buffer [4,13]. Samples were equilibrated at 20 °C for 1 h before the start of experiments.

Prepared chocolate-flavoured MPCs were heated at 90 °C for 5 min or 121 °C for 2.6 min at a constant shear rate (100 or 1000 s^−1^) in a pressure cell (CC25/PR-150) of a rheometer (MCR 302e, Anton Paar GmbH, Graz, Austria) with a constant pressure of 250 kPa following the method of [14]. The heating was accomplished at a rate of 5 °C min^−1^ to the required temperature, followed by the required holding time and cooled down at a rate of 5 °C min^−1^. Shear stress and viscosity were assessed at each treatment condition and the Reynolds number (Re) was calculated as reported previously [15]. The flow was characterised as the laminar for all conditions applying these equations. 

The pH of each treated sample was measured immediately after treatment using a pH meter (WTW Inolab pH 720, Weilheim, Germany) at 20 °C and the first stable endpoint was recorded. A portion of the treated sample was centrifuged (Beckman Optima L-70 Ultracentrifuge, Indianapolis, IN, USA) at 100,000× *g* for 1 h at 20 °C. The supernatant was carefully removed and used for sodium dodecyl sulphate polyacrylamide gel electrophoresis (SDS-PAGE) analysis.

### 2.3. Rheological Measurements

The rheological data of untreated and rheometer-processed protein dispersions were collected using the above-used MCR 302e Rheometer (Anton Paar GmbH, Graz, Austria) and double-gap-cylinder measuring system (DG26.7-SN7721, Anton Paar, Graz, Austria). The viscosity measurements of the dispersions were collected via a programmed logarithmic shear rate ramp, increasing from 0.1 to 1000 s^−1^ for 50 points at 0.5 min intervals at 20 °C. The flow behaviour was described by the Ostwald de Waele model (τ = Kγ^n^), where τ presents shear stress (Pa) and γ is shear rate (s^−1^), while K and n are a consistency factor (Pa·s^n^) and the flow behaviour index, respectively. 

### 2.4. Particle Size Measurements

Immediately after the treatment, particle size measurements were conducted using a Zetasizer (Zetasizer Nano ZS, Malvern Instruments, Malvern, UK), following the methodology outlined by [14]. Prior to the measurements, the treated samples were diluted 1000 times using skim milk ultra-filtrate prepared via the ultrafiltration of milk at 15 °C. This process employed a SEPA CF membrane module (Sterlitech Corporation, Kent, WA, USA) with a polyethersulfone (PES) membrane (190 × 140 mm) and a molecular cut-off of 10 kDa. The refractive indexes used in the calculations were 1.57 for MPC and 1.34 for SMUF, as previously reported [16].

### 2.5. Fourier Transform Infrared (FTIR) Analysis

The changes in the secondary structure of proteins were evaluated using an FTIR spectrometer (PerkinElmer Frontier FTIR Spectrometer, Waltham, MA, USA). FTIR spectra were acquired at room temperature (~20 °C) within 10 min after each treatment. Each spectrum was obtained by averaging 16 scans with a resolution of 4 cm^−1^ after subtracting the background [17]. To enhance resolution for qualitative analysis, the second derivative of all FTIR spectra within the broad Amide I region of 1700–1600 cm^−1^ was calculated using a PerkinElmer software (Spectrum 10 STD). Fourier self-deconvolution (FSD) and baseline correction were performed using an Origin Pro 2018 software (Origin Lab Corporation, Northampton, MA, USA) to identify prominent peaks corresponding to protein secondary structure within Amide I region. Peak fitting was carried out using the Gaussian function and peak fitting method, optimizing the fitting through iterative processes. The areas of the identified prominent peaks associated with specific secondary structures were summed and divided by the total area. This allowed for the determination of peak areas corresponding to five major protein secondary structures: α-helices (1660–1650 cm^−1^), β-sheets (1637–1610 cm^−1^ and 1696–1680 cm^−1^), random coils (1648–1638 cm^−1^), and β-turns (1679–1667 cm^−1^) [18,19]. The obtained results were then subjected to statistical analysis following the guidelines outlined in Section 2.7.

### 2.6. Sodium Dodecyl Sulphide Polyacrylamide Gel Electrophoresis (SDS PAGE)

After the treatments, all the treated samples, control samples, and the supernatants obtained from centrifuged samples (as mentioned earlier) were mixed with sodium dodecyl sulphate (SDS) sample buffer at a ratio of 1:25 (*v*/*v*). These mixtures were then stored at −20 °C until the time of electrophoresis. Both non-reducing and reducing SDS-PAGE (with β-mercaptoethanol as the reducing agent) were conducted following previously described methods [20]. Gel images were captured using Image Lab 5.1 software (Bio-Rad Laboratories, Galesville, NSW, Australia). The intensity of proteins in the treated samples, as observed in the supernatants through reducing gel electrophoresis, was expressed as a percentage of their corresponding proteins present in the control bulk dispersions.

### 2.7. Inductively Coupled Plasma Emission Spectrometric (ICP-OES) Analysis

For the determination of non-sedimentable minerals, samples were prepared by dissolving the ash obtained after combustion in a muffle furnace at 550 °C. This ash was dissolved in a mixture of 10 mL of 1 M HNO_3_ acid and water to achieve a total solid (TS) content of 0.1%. Additionally, five standard solutions with varying concentrations of Ca, Mg, K, and P, ranging from 0.02% to 1% (*w*/*w*), were prepared following the method outlined in [21]. The non-sedimentable mineral content of both these standards and the supernatants after each treatment was then analysed using an inductively coupled plasma (ICP) atomic emission spectrometer (ICP E Multitype, Shimadzu Corporation, Kyoto, Japan), according to the procedure described by [22]. 

### 2.8. Statistical Analysis

Each experiment was performed in triplicate for both the control and treated chocolate-flavoured MPC dispersions, and statistical analyses were conducted using IBM SPSS Statistics software (version 28.0.1.0, IBM Corp., Armonk, NY, USA), employing a multivariate general linear model (GLM) protocol. The concentration of κ-carrageenan, temperature/time combinations, and shearing were considered the main factors in the analysis. The significance level was set at *p* ≤ 0.05. Tukey’s studentized range (HSD) test was used post hoc for multi-comparison of the means.

## 3. Results

### 3.1. Particle Size Distribution and Zeta Potential of MPC Suspensions upon Addition of κ-Carrageenan at Different Concentrations 

The control dispersion had an average particle size of ~227 nm at 20 °C and decreased upon heating up to ~166 nm at 121 °C (Table 1). However, no significant change in zeta potential was observed (Table 1). 

**Figure 1:**
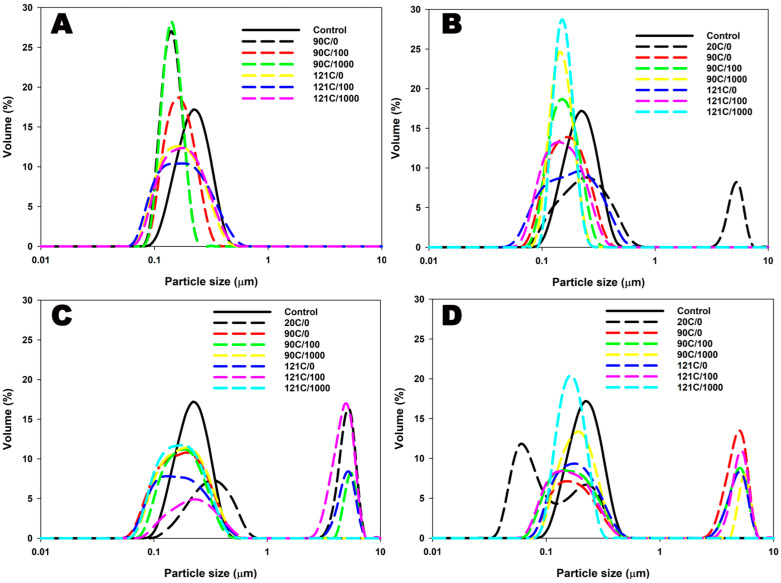
Particle size distribution of chocolate flavoured MPC suspensions without κ-carrageenan (**A**) and with κ-carrageenan concentrations of 0.01% (**B**), 0.03% (**C**), and 0.05% (**D**) processed under different temperatures (20, 90, or 121 °C) and shear rates (0, 100, or 1000 s^−1^).

The addition of κ-carrageenan to the MPCs at 20 °C resulted in an increase in average particle size from ~227 nm at the initial to ~297 nm at 0.05% κ-carrageenan concentration (Table 1). This size change can be attributed to the adsorption of κ-carrageenan onto the surface of casein micelles during the initial preparation of protein–polysaccharide mixtures at an elevated temperature [23]. Furthermore, the addition of κ-carrageenan also induced a transition in the particle size distribution from monomodal to bimodal (Figure 1). This shift was characterized by a particle population that is relatively larger compared to dispersions without κ-carrageenan and appeared only at low and intermediate κ-carrageenan concentrations. The resultant particles were observed within the size range of both 0.1–1 μm and >1 μm. This could be indicative of a creation of a localised κ-carrageenan network that would entrap casein micelles [24], thus giving the appearance of larger particles. The zeta potential became more negative, from ~−20.8 mV in the absence of κ-carrageenan to ~−28 mV at 0.01% κ-carrageenan. When the concentration of κ-carrageenan was increased, zeta potential declined (e.g., ~−23.5 mV at 0.05% κ-carrageenan concentration) (Table 1). At low concentrations, κ-carrageenan may adsorb onto the surface of casein micelles, leading to an increase in the negative charge [23,24]. As κ-carrageenan concentrations increase, a network of κ-carrageenan molecules may form, trapping casein micelles within the network and resulting in a decreased negative charge and zeta potential [23].

Particle sizes in heated dispersions with added κ-carrageenan decreased across all concentrations (e.g., from ~297 nm at 20 °C to ~182 nm at 121 °C at 0.05%). However, the resulting particles were comparatively larger than particles without the stabilizer (Table 1). This change occurred in a concentration-dependent manner, with higher concentrations leading to larger particles (Figure 1 and Table 1). Consequently, zeta potential values also became less negative in all heated dispersions.

When subjected to combined heating and shearing, no significant changes in particle size or zeta potential were observed in control dispersions without κ-carrageenan (Figure 1A and Table 1). However, the addition of κ-carrageenan to the dispersions and subsequent exposure to heat and shear resulted in a more uniform size distribution of particles, with an increase in the average particle size as the shear rate increased (e.g., ~182 nm at 0 s^−1^ to ~198 nm at 1000 s^−1^ at 121 °C and at 0.05%) (Figure 1 and Table 1). Particles ranging between 0.1 and 1 μm were observed in MPCs without κ-carrageenan. When the shear rate was increased up to 1000 s^−1^ in the dispersions with added κ-carrageenan, the proportion of particles within the size range of 0.1–1 μm was increased compared to 100 s^−1^ (Figure 1). In contrast, at 100 s^−1^, there was a relatively larger proportion of particles observed >1 μm. This suggests that at 1000 s^−1^, a higher number of casein particles did not interact with κ-carrageenan or aggregates formed were fragmented due to shear, while at 100 s^−1^, more interactions occurred between caseins and κ-carrageenan. This implies that the interaction between casein micelles and κ-carrageenan may be influenced by the applied shear rate (Figure 1 and Table 1). 

Specifically, at a shear rate of 1000 s^−1^, aggregates within the larger particle size range were considerably disrupted, leading to the generation of particles with more uniform sizes. This effect was particularly pronounced at lower concentrations of κ-carrageenan (0.01% and 0.03%) and at a temperature of 121 °C (Figure 1 and Table 1). At elevated concentrations, κ-carrageenan has the ability to create a robust polysaccharide network in which casein micelles become physically entrapped [24,25]. However, the stability of this κ-carrageenan network is influenced by the amount of κ-carrageenan present. When subjected to cooling, the shear forces applied can disrupt the formation of the κ-carrageenan network, leading to its destabilization and potential breakdown. 

### 3.2. Mineral Distribution of MPCs upon Addition of κ-Carrageenan at Different Concentrations

Ca, Mg, K, and P collectively constitute a significant fraction of mineral constituents in milk. These ions exhibit diverse associations among themselves and with milk proteins. Notably, K demonstrates full solubility, whereas Ca, Mg, and P exhibit partial association with casein micelles [26]. The partitioning of these elements between non-sedimentable and micellar phases markedly influences the stability of the colloidal dispersion in milk [27].

The control dispersion at 20 °C contained ~5.84 mM of non-sedimentable calcium. Heating control dispersions primarily resulted in a decrease in the levels of non-sedimentable calcium up to ~4.96 mM at 121 °C, as it precipitated as calcium phosphate due to the applied heat (Table 2). However, this reduction was not as prominent as in κ-carrageenan-added dispersions. 

As depicted in the table, Mg content within the MPC suspensions exhibited notable elevation, likely attributable to the inclusion of cocoa [28]. Upon the addition of κ-carrageenan at 20 °C, there was a significant reduction in the levels of non-sedimentable minerals, including Ca, Mg, and P (Table 2). Furthermore, as the concentration of κ-carrageenan increased, these mineral levels within the supernatant decreased even further (e.g., Ca content decreased from ~5.84 mM at 20 °C in the absence of κ-carrageenan to ~2.37 mM at 0.05% concentration of κ-carrageenan) (Table 2). This reduction in non-sedimentable mineral content can be attributed to the interaction between the κ-carrageenan and minerals in milk and interactions with proteins, which can impact the distribution and availability of minerals within the dispersions. This effect appeared to be augmented with the application of heat, as it further reduced Ca, K, and P from the soluble phase, likely due to heat-induced precipitation or involvement in the formation of a CN/κ-carrageenan network (e.g., Ca content was further decreased from ~2.37 nm at 20 °C to ~1.97 nm at 121 °C and at 0.05%) [29]. 

The application of combined heat and shear to the dispersions without κ-carrageenan resulted in an increase in all the investigated minerals in the soluble phase, including Ca, K, Mg, and P with a comparatively greater incline in both Ca and K (Table 2). In the dispersions containing κ-carrageenan, the impact of shear on minerals appears to be dependent on the concentration of κ-carrageenan at both temperatures. At a low concentration of κ-carrageenan (0.01%), the combined application of heat and shear resulted in an increase in all non-sedimentable minerals with a >50% increase in Ca, K, and P levels at 1000 s^−1^ and at 121 °C (Table 2). However, as the concentration of κ-carrageenan increased, there was either a slight decline or no substantial change in the levels of non-sedimentable minerals (Table 2). The decline in minerals within the soluble phase may be attributed to the enhanced stability of the CN-κ-carrageenan network formed with the involvement of minerals in the presence of higher κ-carrageenan concentrations, which is less susceptible to disruption by shear forces.

### 3.3. Rheological Properties of MPCs upon Addition of κ-Carrageenan at Different Concentrations

Both non-heated and heated control dispersions display shear-thinning behaviour (characterised by *n* < 1), signifying that apparent viscosity decreases as shear rate increases. With heating, the consistency factor decreased, leading to lower apparent viscosity (Table 3). 

**Figure 2:**
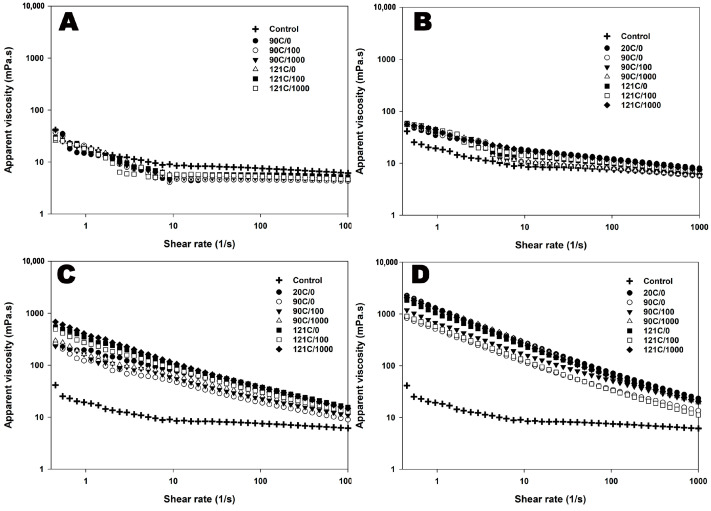
Apparent viscosity of chocolate flavoured MPC suspensions as a function of shear rate (0, 100, or 1000 s^−1^) applied during heating at 90 °C for 5 min or 121 °C for 2.6 min at 0% κ-carrageenan (**A**), 0.01% κ-carrageenan (**B**), 0.03% κ-carrageenan (**C**), or 0.05% κ-carrageenan (**D**). The viscosity measurements were completed after the treatments at 20 °C.

In the presence of κ-carrageenan, all dispersions exhibited shear-thinning behaviour, regardless of the treatment they underwent, as indicated by *n* < 1 in all κ-carrageenan-added dispersions. The addition of κ-carrageenan resulted in an increase in apparent viscosity, as evidenced by an elevated consistency factor (Figure 2 and Table 3). This increase in viscosity can be attributed to the linear macromolecular structure and polyelectrolytic properties of carrageenan, which promotes interactions with proteins and minerals. Consequently, apparent viscosities were observed to increase with higher κ-carrageenan concentrations. The highest apparent viscosity was observed at a κ-carrageenan concentration of 0.05% (Table 3). When dispersions containing added κ-carrageenan were heated, a decrease in the consistency factor was observed across all three concentrations (Table 3). This decrease was notably more pronounced after treatment at 90 °C compared to 121 °C.

As observed with non-heated and heated controls, the combined application of heat and shear to controls resulted in the system that exhibited shear-thinning behaviour (indicated by *n* < 1) with an increase in the consistency factor as shear rate increased, signifying an increase in viscosity (Figure 2 and Table 3). Similarly, all κ-carrageenan-added dispersions exhibited shear-thinning behaviour (*n* < 1) under the influence of combined heat and shear. The changes in the consistency factor were dependent on the concentration of κ-carrageenan. In a general trend, elevated temperatures and increased shear rates led to higher consistency factors, consequently elevating the apparent viscosity of all κ-carrageenan-added dispersions (Figure 2 and Table 3). At higher concentrations of κ-carrageenan, the formation of a more robust κ-carrageenan–casein network, resistant to shear forces, ultimately contributed to the increased apparent viscosity of these dispersions. 

### 3.4. Interactions and Aggregation of Proteins as Observed by SDS-PAGE Analysis upon the Addition of κ-Carrageenan at Different Concentrations

In the control dispersions, an increase in temperature resulted in a decline of β-LG and α-LA levels in the soluble phase due to aggregation and precipitation. On the other hand, all α_s_-, β-, and κ-CN levels increased in the soluble phase, depicting the dissociation of caseins with the increase in temperature (Supplementary Materials, Figure S1 and Table 4).

In the dispersions containing added κ-carrageenan, it was observed that the levels of both whey proteins and caseins in the serum phase decreased with increasing κ-carrageenan concentration at 20 °C, consistent with findings from previous studies (Supplementary Materials, Figure S1 and Table 4) [30]. This can be attributed to the formation of a protein-κ-carrageenan network, which sedimented upon ultracentrifugation.

Heating MPCs with added κ-carrageenan substantially increased the levels of non-sedimentable caseins (particularly κ-CN), probably due to detachment of κ-CN/κ-carrageenan complexes from casein micelles and the destabilisation of micelles. However, soluble whey proteins in the supernatant declined further, probably due to heat-induced denaturation and aggregation (Supplementary Materials, Figure S1 and Table 4). 

In the dispersions without added κ-carrageenan, the combined application of heat and shear gradually increased the levels of all β-LG, α-LA, and κ-CN in the soluble phase compared to heated dispersions at both 90 and 121 °C. This increase indicates the shear-induced fragmentation of aggregates. Additionally, both α_s_- and β-CN levels in the soluble phase also increased with a notably higher concentration of β-CN, reaching their highest levels at 1000 s^−1^ and 121 °C, likely due to the shear-induced structural destabilization of caseins (Supplementary Material Figure S1 and Table 4). On the other hand, in all κ-carrageenan-added dispersions, and the combined application of heat and shear decreased the levels of all β-LG, α-LA, and κ-CN in the soluble phase, indicating the precipitation of these proteins upon centrifugation. All these proteins appeared to decrease in a concentration-dependent manner, reaching their lowest levels at a κ-carrageenan concentration of 0.05% (Supplementary Materials, Figure S1 and Table 4). 

### 3.5. Conformational Properties of MPC System with Added k-Carrageenan

**Figure 3:**
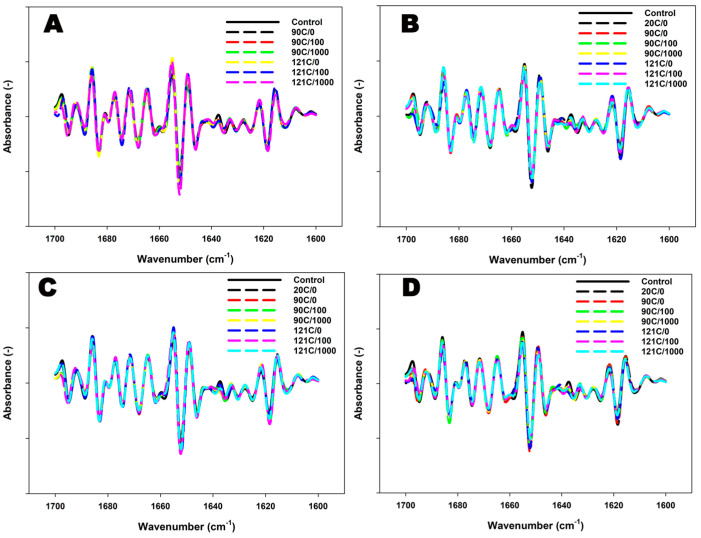
FTIR spectra (second derivative) for the Amide I region of chocolate-flavoured MPC suspensions without κ-carrageenan (**A**) and with κ-carrageenan concentrations of 0.01% (**B**), 0.03% (**C**), and 0.05% (**D**) processed under different temperatures (20, 90, or 121 °C) and shear rates (0, 100, or 1000 s^−1^).

In the control dispersions, heating resulted in a notable reduction in the peak intensity at ~1637 cm^−1^ (Figure 3) and a significant decrease in its content (Table 5), indicating a decline in intramolecular β-sheets. Furthermore, prominent peaks at ~1671 cm^−1^ and ~1674 cm^−1^ were observed (Figure 3) with a significant increase in their peak areas (Table 5), suggesting a concurrent increase in β-turns.

The addition of κ-carrageenan at 20 °C resulted in reduced peak intensities, particularly at ~1655 cm^−1^ and ~1637 cm^−1^, and reduced peak areas, indicating a decrease in α-helical structures and intramolecular β-sheets in a concentration-dependant manner in the MPC system, respectively (Figure 3, Table 5). Simultaneously, an increase in random coil structures was observed, represented by intense peaks at ~1642 cm^−1^ and its content (Figure 3, Table 5). Heating of κ-carrageenan-added dispersions led to a further reduction in peak intensity at both ~1655 cm^−1^ and ~1637 cm^−1^ (Figure 3), along with a decrease in their content (Table 5), indicating a decline in α-helical structures and intramolecular β-sheets, respectively. Simultaneously, an increase in peak intensities at ~1672 cm^−1^ was observed, suggesting an increase in β-turns. These changes appeared to be moderated with the further addition of κ-carrageenan (Figure 3 and Table 5).

The combination of heat and shear applied to MPCs without added κ-carrageenan further reduced intramolecular β-sheets while increasing β-turns, as indicated by the reduction in peak intensities at ~1637 cm^−1^, ~1622 cm^−1^, and their respective content, along with an increase in peak intensity at ~1672 cm^−1^ and its content (Figure 3 and Table 5). Additionally, a decline in α-helix content and a simultaneous increase in random coils were also observed (Table 5). Furthermore, the aggregated β-sheets were also decreased, as observed (Figure 3, Table 5). The combined application of heat and shear to κ-carrageenan-added dispersions resulted in a reduction in peak intensity at ~1637 cm^−1^ and its content, depicting a gradual decline in intramolecular β-sheets. There was an increase in peak intensity within the ~1682–1700 cm^−1^ region in all κ-carrageenan-added dispersions, along with its content, depicting an increase in aggregated β-sheets. The number of β-turns gradually declined as observed in the reduction in peak intensity at ~1672 cm^−1^ and its content (Figure 3 and Table 5). This decrease was most prominent at the highest concentration of κ-carrageenan. Additionally, there was a reduction in peak intensity at ~1655 cm^−1^, along with its content, indicating a reduction in α-helical structures (Figure 3 and Table 5). Simultaneously, an increase in random structures was also observed along with the increased heat and shear (Table 5).

## 4. Discussion

### 4.1. Effect of κ-Carrageenan Concentration on Chocolate-Flavoured MPC Dispersions at 20 °C

Under neutral pH conditions (~pH 6.8), an electrostatic interaction occurs between κ-carrageenan and κ-CN at the surface of the casein micelles [24,30]. Specifically, a segment of κ-CN spanning residues 97 to 112 plays a significant role in these interactions, exhibiting a considerable positive charge capable of establishing an electrostatic bond with the negatively charged sulphate groups present in κ-carrageenan [31,32]. κ-Carrageenan is composed of linear polymers that have a backbone structure made up of alternating α-1,4- and β-1,3-linked galactose residues. These polymers also contain different amounts of sulphate half-ester groups, providing a negative charge and playing a role in shaping their properties and functions [33]. Cations such as Ca can also serve as bridges between the carboxyl groups on proteins (e.g., milk protein or proteins from cocoa) and the sulphate groups on carrageenan [34]. As a result, a reduction in soluble minerals such as Ca and Mg were observed alongside the κ-carrageenan concentration [29,35]. Similar interactions involving carboxyl and sulphate groups can also occur in the case of whey proteins at neutral pH [36,37]. This was supported by the FTIR results as alterations observed in α-helical structures along with the potential unfolding of intramolecular β-sheets into more random structures following the addition of κ-carrageenan, indicating their involvement in these interactions. This interplay contributes to the decline in soluble caseins and whey proteins, as observed in SDS PAGE data, a consequence of their interactions with κ-carrageenan as its concentration ascends. 

At 20 °C and particularly at lower concentrations, κ-carrageenan serves as an effective stabilizer, preventing the agglomeration of particles in the milk dispersion. The elevation of κ-carrageenan concentration at 20 °C induces the extension of carrageenan molecules, facilitated by the mutual repulsion arising from the abundance of negatively charged half-ester sulphate groups along the polymer chain [38]. Moreover, owing to its hydrophilic nature, the carrageenan molecules become enveloped by a sheath of immobilized water molecules [38]. As the κ-carrageenan concentration increases, the enhanced suspension and stability of milk particles emerges through the formation of a protective barrier enveloping the particles [39]. Additionally, milk dispersions mixed with κ-carrageenan at 20 °C exhibits more heterogeneity due to areas rich in carrageenan and other areas rich in casein micelles [40]. Consequently, these factors contribute to increased particle size. The formation of loosely associated fractal structures following the addition of κ-carrageenan facilitates the immobilization of water within the dispersant. This process augments the effective volume fraction, consequently increasing apparent viscosity [41]. Additionally, the higher solids content contributes to increased viscosity through molecular movements and interfacial film formation [42,43].

### 4.2. Effect of Heating on Chocolate-Flavoured MPC Dispersions with Varying Levels of κ-Carrageenan

κ-Carrageenan exhibits different conformations depending on the temperature. At elevated temperatures (>50 °C and depending on ion concentration), it exists as a random coil in solution. However, as the temperature decreases, it undergoes a conformational transition from a coil to a helix structure (~37 °C). This helix formation and subsequent aggregation of neighbouring helices promote the gelation process [44,45]. In addition, κ-carrageenan becomes more accessible when it transitions from random coil structures to helices upon binding with positively charged κ-CN [32]. The helical conformation of κ-carrageenan leads to a higher charge density as the sulphate groups come closer together, facilitating electrostatic interactions with casein [46,47]. Furthermore, this helical conformation promotes the sequestration of minerals within these sulphate groups, ultimately leading to reduced levels of minerals in the serum phase. Studies utilizing dynamic light scattering (DLS) have demonstrated that the presence of κ-carrageenan causes an increase in the diameter of casein micelles as the temperature drops below the coil–helix transition temperature [31]. This increase in diameter suggests adsorption and molecular interaction between κ-carrageenan and casein. Thus, it becomes evident that the incorporation of κ-carrageenan results in a gradual enlargement of particle size (Table 1). However, this trend reverses upon heating. One of the reasons could be the homogenizing effect caused by thermodynamic influences [40]. Another reason could be the micellar disaggregation, in which κ-CN/κ-carrageenan complexes detach from the casein micelles at elevated temperatures, as evidenced by a substantial increase in κ-CN in the soluble phase upon heating (Table 3), resulting in a reduced particle size (Table 1) [48]. On the other hand, in the absence of κ-carrageenan, the interaction of cocoa components, such as polyphenols, with casein proteins can potentially modify their structure and stability of milk proteins, as observed in the results [49]. This interaction, combined with the heating process, further promotes the breakdown of casein micelles into smaller particles, resulting in decreased particle size [49]. 

The influence of milk proteins was found to be dominant when carrageenan concentration was <0.018% *w*/*w* in milk [33]. Under these conditions, milk proteins interfere with gel formation leading to the domination of protein–carrageenan interactions, reducing the availability of carrageenan molecules in a gelation role. Additionally, a substantial component of protein–protein interactions occurred due to low levels of carrageenan. At high levels of carrageenan (>0.01%), milk proteins have a minimal apparent effect on gelation. Therefore, the association of κ-carrageenan helices resulting in the formation of robust self-supporting gels that entrapped both casein micelles and casein micelle–carrageenan complexes. [33]. Therefore, it is clear that changes in secondary structures were more pronounced at lower κ-carrageenan levels, as interruptions by κ-carrageenan were minimal, as mentioned above, allowing for the increased availability of caseins for potential interactions [50].

In addition to the concentration of κ-carrageenan, the influence of heat can profoundly impact the resulting gelation and apparent viscosity. A higher temperature appears to be required to activate the carrageenan network, as evidenced by the higher consistency factor at 121 °C compared to that at 90 °C depicting a stronger gelation process. The carrageenan molecules may not fully hydrate or transition and interact with proteins and each other, resulting in a less organized and weaker gel structure at 90 °C. In contrast, the higher temperature (121 °C) facilitates the better hydration and transition of the carrageenan molecules, leading to a more organized and tightly bonded gel network. This stronger gel structure can trap more water and milk components, resulting in a higher apparent viscosity of the milk dispersions with κ-carrageenan at 121 °C. Furthermore, as previously mentioned, the detachment of κ-CN/κ-carrageenan complexes at elevated temperatures results in an increase in particle concentration, consequently leading to a rise in consistency factor, and thereby, apparent viscosity. Moreover, it is apparent that the influence of heat is dependent upon the concentration of κ-carrageenan. Specifically, at 0.05% κ-carrageenan concentration, the particle size distribution at both 90 °C and 121 °C underwent a transition from a monomodal to a bimodal distribution. This alteration signifies a relatively larger fraction of particles exceeding the >1 μm range and is indicative of a more pronounced network formation at both temperatures. This observation is substantiated by the heightened consistency factor observed at 0.05% κ-carrageenan concentration in contrast to other dispersions containing κ-carrageenan, thereby resulting in an increased apparent viscosity.

The impact of whey protein denaturation on κ-carrageenan’s gelation abilities in milk was studied [6,51], and it was determined that it did not substantially affect κ-carrageenan’s gelation. Additionally, the κ-carrageenan present in solution during heat treatment of milk did not have a notable effect on the rate of whey protein denaturation. In fact, the formation of the β-LG/κ-CN complex entails a sulphydryl-disulphide interchange mechanism between the sulphydryl groups on β-LG and the cysteinyl residues located in the hydrophobic domain of κ-CN. Conversely, the interaction between κ-CN and κ-carrageenan is mediated by the glycopeptide region found within the hydrophilic domain of κ-CN and the negatively charged sulphate groups present on the carrageenan molecule [48]. However, the heat-induced denaturation of β-LG may expose more positively charged groups, facilitating electrostatic interactions with the sulphate groups of κ-carrageenan, ultimately leading to the formation of complexes [52,53]. Hence, heating resulted in a reduction of whey proteins, as evidenced by PAGE analysis, attributed to heat-induced denaturation and subsequent aggregation with other proteins, potentially including κ-carrageenan. On the other hand, α_s_-CN, β-CN, and κ-CN levels exhibited a concentration-dependent increase within the soluble phase along with the heating (Table 4). 

### 4.3. Effect of Combined Heat and Shear on Chocolate-Flavoured MPC Dispersions with Varying Levels of κ-Carrageenan

The combined application of heat and shear to milk proteins can lead to a cascade of changes, including denaturation, unfolding, aggregation, the fragmentation of large aggregates, and altered interactions with other components, such as minerals and polysaccharides [10,54,55]. Higher concentrations of κ-carrageenan, in conjunction with shear forces, led to an increase in average particle size and a reduction in non-sedimentable mineral content. This growth in particles in a dispersion due to shear can be attributed to several underlying factors. First, the heightened shear and pressure resulting from rotational diffusion, due to a tumbling motion, facilitated rapid particle association through a combination of hydrophobic and electrostatic interactions [56,57]. Furthermore, the periodic tumbling motion induced by the high shear rates in the fluid flow field may introduce conformational distortions in the molecules, leading to kinked states. These kinked states, in turn, promote the gradual unravelling of molecules and enhance intermolecular hydrodynamic interactions [58,59]. Additionally, molecules brought into proximity via hydrophobic interactions are likely to engage in disulphide bridging during the heating process. This occurs due to the exposure of previously concealed free sulfhydryl groups during the unfolding of molecules [14]. The observed increase in the average particle size at 1000 s⁻^1^ at higher concentration of κ-carrageenan is substantiated by notable reductions in the levels of κ-CN, αs-CN, and β-CN, as well as both β-LG and α-LA in the serum phase. This reduction strongly suggests their involvement in the overall aggregation, as supported by the SDS-PAGE data. Notably, a more pronounced reduction in β-LG, α-LA, and κ-CN in a concentration-dependent manner implies an increased propensity for aggregation among these specific proteins and with κ-carrageenan under the influence of shear. In addition, as indicated by FTIR data, the gradual decline in intramolecular β-sheets and incline in aggregated β-sheets, when the combined heat and shear was applied to κ-carrageenan-added dispersions, depicts the shear-induced aggregation. The number of β-turns declined as a result of reduced free caseins within the soluble phase. This decrease was most prominent at the highest concentration of κ-carrageenan, owing to the comparatively low availability of caseins due to the entrapment of these in a more robust gel network [60]. The aggregation induced by shear forces, leading to the formation of larger particles, can result in a heightened resistance to flow, consequently prompting an increase in the consistency factor and thereby the apparent viscosity of suspensions (Table 3).

## 5. Conclusions

The impact of shear on chocolate-flavoured milk dispersions appears primarily dependent on the concentration of κ-carrageenan and heating temperature. At higher carrageenan concentrations, gelation is predominantly driven by interactions among carrageenan molecules themselves, resulting in the entrapment of casein micelles and carrageenan–casein micelle complexes. As a result, the intense destabilization of casein micelles and increased collision rates under shear conditions lead to further aggregation, reducing the presence of both caseins and whey proteins in the soluble phase. Simultaneously, this process increases the average particle size and apparent viscosity within the dispersions.

Given that these processing conditions are commonly encountered in production, it is essential to carefully consider them during food processing to achieve the desired product characteristics while minimizing any potential adverse effects.

## Figures and Tables

**Table 1:** Average particle size and zeta-potential of MPC suspensions with adjusted κ-carrageenan (κ-CG) concentrations subjected to different temperatures (20, 90, or 121 °C) and shear rates (0, 100, or 1000 s^−1^).

κ-CG (%)	Temp. (°C)	Average Particle Size (nm)			Zeta Potential (mV)		
		0 s^−1^	100 s^−1^	1000 s^−1^	0 s^−1^	100 s^−1^	1000 s^−1^
0	20	227 ^C^			−20.8 ^AB^		
	90	159 ^Gb^	154 ^Eb^	163 ^Ea^	−24.4 ^BCDa^	−22.8 ^Ca^	−22.5 ^Aa^
	121	166 ^FGb^	171 ^Da^	171 ^Da^	−22.5 ^ABCa^	−23.9 ^Ca^	−22.0 ^Aa^
0.01	20	211 ^D^			−27.9 ^D^		
	90	177 ^EFa^	179 ^Da^	185 ^Ca^	−21.5 ^ABCa^	−21.6 ^BCa^	−20.9 ^Aa^
	121	172 ^EFGb^	199 ^Ca^	173 ^Db^	−20.5 ^Aa^	−17.5 ^Aa^	−20.0 ^Aa^
0.03	20	269 ^B^			−24.6 ^CD^		
	90	179 ^EFb^	197 ^Ca^	167 ^Dc^	−20.4 ^Aa^	−21.6 ^BCa^	−23.3 ^Aa^
	121	177 ^EFc^	226 ^Aa^	209 ^Ab^	−23.1 ^ABCa^	−18.5 ^ABa^	−21.4 ^Aa^
0.05	20	297 ^A^			−23.5 ^ABC^		
	90	185 ^Ec^	205 ^Ba^	193 ^Bb^	−22.6 ^ABCb^	−21.7 ^BCa^	−21.0 ^Aa^
	121	182 ^EFc^	203 ^Ba^	198 ^ABa^	−24.8 ^CDb^	−22.9 ^Cab^	−21.9 ^Aa^
SEM *		2.57			−0.70		

The means in a column with different superscript upper-case letters and in a row with different superscript lower-case letters differ significantly (*p* < 0.05). * Standard error of mean.

**Table 2:** Mineral concentration in supernatants of MPC suspensions with adjusted κ-carrageenan concentrations subjected to different temperatures (20, 90, or 121 °C) and shear rates (0, 100, or 1000 s^−1^).

κ-CG (%)	Temp (°C)	Mineral Concentration (mM)											
		Ca			K			Mg			P		
		0 s^−1^	100 s^−1^	1000 s^−1^	0 s^−1^	100 s^−1^	1000 s^−1^	0 s^−1^	100 s^−1^	1000 s^−1^	0 s^−1^	100 s^−1^	1000 s^−1^
0	20	5.84 ^A^			10.19 ^D^			17.05 ^A^			7.47 ^A^		
	90	5.76 ^Ac^	7.45 ^Ab^	7.85 ^Aa^	9.27 ^Eb^	9.61 ^Db^	10.58 ^Ea^	16.54 ^Ab^	18.10 ^Aa^	18.33 ^Aa^	6.84 ^ABb^	9.06 ^Aa^	9.23 ^Aa^
	121	4.96 ^Bc^	6.68 ^Bb^	7.29 ^Ba^	8.97 ^Fb^	7.79 F^c^	14.73 ^Aa^	16.00 ^Bc^	17.21 ^Bb^	18.57 ^Aa^	6.64 ^ABb^	8.67 ^Aa^	8.30 ^Ba^
0.01	20	4.08 ^C^			12.87 ^A^			14.56 ^C^			6.26 ^ABC^		
	90	3.70 ^Cb^	3.75 ^Db^	3.83 ^Db^	10.62 ^Cc^	11.10 ^Bb^	12.40 ^Ba^	14.93 ^Cb^	15.15 ^Dab^	15.60 ^Ca^	4.87 ^DEb^	5.59 ^Ca^	6.10 ^Ca^
	121	3.11 ^Db^	4.82 ^Ca^	4.81 ^Ca^	6.31 ^Gc^	9.40 ^Db^	10.19 ^Fa^	16.58 b	16.58 ^Cb^	17.22 ^Ba^	4.57 ^DEc^	6.38 ^Bb^	8.53 ^ABa^
0.03	20	3.70 ^C^			11.30 ^B^			13.79 ^D^			6.06 ^BCD^		
	90	3.60 ^Ca^	3.42 ^Dab^	2.92 ^Eb^	12.84 ^Aa^	10.21 ^Cc^	11.18 ^Cb^	16.93 ^Aa^	16.54 ^Cab^	10.21 ^Cb^	5.80 ^CDa^	5.02 ^Db^	5.35 ^Cb^
	121	2.63 ^Eb^	3.01 ^Da^	2.80 ^Eab^	12.58 ^Aa^	12.02 ^Ab^	10.79 ^Dc^	14.54 ^Cb^	15.07 ^Da^	14.44 ^Db^	5.92 ^BCDa^	5.89 ^Ca^	5.37 ^Cb^
0.05	20	2.37 ^EF^			12.23 ^A^			13.67 ^D^			5.01 ^CDE^		
	90	2.34 ^EFa^	2.30 ^Ea^	2.10 ^Fa^	11.58 ^Ba^	11.19 ^Bab^	10.87 ^Db^	14.65 ^Cb^	15.38 ^Da^	14.60 ^Db^	5.06 ^CDEa^	4.87 ^Ea^	4.21 ^Db^
	121	1.97 ^Fa^	1.77 ^Fa^	1.56 ^Ga^	9.52 ^Ea^	8.70 ^Eb^	8.23 ^Gc^	13.93 ^Da^	12.85 ^Eb^	12.49 ^Eb^	3.84 ^Ea^	3.14 ^Fb^	2.22 ^Ec^
SEM *		0.05			0.08			0.12			0.07		

The means in a column with different superscript upper-case letters and in a row with different superscript lower-case letters differ significantly (*p* < 0.05). * Standard error of mean.

**Table 3:** Consistency factor (K) and flow behaviour index (n) of MPC suspensions with adjusted κ-carrageenan concentrations subjected to different temperatures (20, 90, or 121 °C) and shear rates (0, 100, or 1000 s^−1^).

κ-CG (%)	Temp. (°C)	Consistency Factor (Pa.s^n^)			Flow Behaviour Index (-)		
		0 s^−1^	100 s^−1^	1000 s^−1^	0 s^−1^	100 s^−1^	1000 s^−1^
0	20	0.011 ^HI^			0.91 ^B^		
	90	0.006 ^IJb^	0.007 ^Gab^	0.008 ^Fa^	0.98 ^Aa^	0.98 ^Aa^	0.97 ^Aa^
	121	0.005 ^Jb^	0.006 ^Ga^	0.007 ^Fa^	0.96 ^Aa^	0.96 ^Aa^	0.95 ^Ba^
0.01	20	0.028 ^G^			0.82 ^D^		
	90	0.014 ^Ha^	0.014 ^Fa^	0.015 ^Ea^	0.87 ^Ca^	0.87 ^Ba^	0.86 ^Ca^
	121	0.021 ^Gb^	0.022 ^Eb^	0.028 ^Da^	0.84 ^Da^	0.84 ^Ca^	0.81 ^Db^
0.03	20	0.170 ^E^			0.65 ^F^		
	90	0.089 ^Fc^	0.103 ^Db^	0.119 ^Ca^	0.67 ^Ea^	0.67 ^Da^	0.67 ^Ea^
	121	0.236 ^Dc^	0.370 ^Bb^	0.641 ^Ba^	0.60 ^Ga^	0.61 ^Ga^	0.50 ^Fb^
0.05	20	0.763 ^A^			0.49 ^I^		
	90	0.243 ^Cc^	0.367 ^Cb^	0.709 ^Aa^	0.58 ^Ga^	0.57 ^Fa^	0.47 ^Gb^
	121	0.622 ^Bc^	0.634 ^Ab^	0.641 ^Ba^	0.51 ^Ha^	0.51 ^Ga^	0.46 ^Fb^
SEM *		0.0017			0.0061		

The means in a column with different superscript upper-case letters and in a row with different superscript lower-case letters differ significantly (*p* < 0.05). * Standard error of mean.

**Table 4:** Intensity of caseins and whey proteins in supernatants as a proportion (%) of their intensity in the control bulk suspensions subjected to different treatments resolved under reducing electrophoretic conditions and quantified using a ChemiDoc imager.

κ-CG (%)	Temp. (°C)	α_s_-CN			β-CN			κ-CN			β-LG			α-LA		
		0 s^−1^	100 s^−1^	1000 s^−1^	0 s^−1^	100 s^−1^	1000 s^−1^	0 s^−1^	100 s^−1^	1000 s^−1^	0 s^−1^	100 s^−1^	1000 s^−1^	0 s^−1^	100 s^−1^	1000 s^−1^
0	20	4.5 ^CD^			8.9 ^CD^			43.6 ^H^			99.5 ^A^			98.9 ^A^		
	90	4.7 ^BCb^	6.4 ^Aa^	6.7 ^Aa^	12.0 ^Bb^	14.5 ^Ba^	14.9 ^Ba^	44.7 ^Gb^	47.7 ^Ea^	48.1 ^Da^	75.3 ^Hb^	80.4 ^Aa^	80.9 ^Aa^	85.3 ^Cc^	88.5 ^Aa^	87.1 ^Ab^
	121	4.8 ^Bb^	5.3 ^Bab^	5.9 ^Aa^	16.4 ^Ac^	17.7 ^Ab^	23.7 ^Aa^	48.3 ^Ec^	51.6 ^Db^	53.8 ^Ba^	68.1 ^Kc^	75.2 ^Cb^	77.2 ^Ca^	63.1 ^Kc^	75.9 ^Cb^	76.8 ^Ba^
0.01	20	4.0 ^E^			8.4 ^D^			32.6 ^I^			97.4 ^B^			94.4 ^B^		
	90	4.7 ^BCa^	4.2 ^Cbc^	3.9 ^Cc^	9.0 ^CDa^	7.7 ^Db^	5.7 ^Dc^	59.6 ^Ba^	57.1 ^Cb^	49.6 ^Cc^	82.6 ^Fa^	80.7 ^Ab^	79.9 ^Bc^	80.1 ^Ea^	80.3 ^Ba^	75.4 ^Bb^
	121	5.3 ^Ab^	5.6 ^Ba^	4.5 ^BCc^	9.7 ^Ca^	8.9 ^Cb^	8.0 ^Cc^	64.4 ^Aa^	46.7 ^Fb^	16.8 ^Hc^	73.5 ^Ia^	66.8 ^Eb^	60.2 ^Gc^	73.6 ^Ga^	53.2 ^Gb^	45.2 ^Gc^
0.03	20	3.6 ^F^			5.5 ^FG^			16.6 ^J^			95.9 ^C^			82.7 ^D^		
	90	4.3 ^Da^	3.9 ^Cb^	3.3 ^Cc^	6.3 ^EFa^	6.1 ^Eab^	5.7 ^Db^	64.4 ^Ab^	66.8 ^Aa^	60.7 ^Ac^	87.3 ^Da^	77.3 ^Bb^	74.8 ^Dc^	77.7 ^Fa^	75.5 ^Cb^	71.0 ^Cc^
	121	5.4 ^Aa^	5.6 ^Ba^	4.9 ^Bb^	7.2 ^Ea^	6.7 ^Eb^	5.9 ^Dc^	53.8 ^Cb^	60.6 ^Ba^	36.6 ^Ec^	75.7 ^Ha^	74.8 ^Cb^	69.5 ^Fc^	66.7 ^Ib^	68.8 ^Da^	64.9 ^Dc^
0.05	20	2.1 ^I^			3.6 ^H^			16.0 ^K^			84.3 ^E^			64.2 ^J^		
	90	2.5 ^Ha^	2.2 ^Eb^	2.0 ^Eb^	4.0 ^Ha^	3.9 ^Ga^	3.1 ^Fb^	47.6 ^Fa^	43.7 ^Gb^	33.2 ^Fc^	79.5 ^Ga^	75.1 ^Cb^	70.4 ^Ec^	71.2 ^Ha^	63.8 ^Eb^	58.9 ^Ec^
	121	3.3 ^Ga^	3.1 ^Dab^	2.7 ^Db^	5.1 ^Ga^	4.9 ^Fa^	4.3 ^Eb^	49.8 ^Da^	42.4 ^Hb^	22.5 ^Gc^	72.1 ^Ja^	67.9 ^Db^	60.7 ^Gc^	62.1 ^La^	55.5 ^Fb^	54.3 ^Fc^
SEM *			0.081			0.278			0.133			0.158			0.163	

The means in a column with different superscript upper-case letters and in a row with different superscript lower-case letters differ significantly (*p* < 0.05). * Standard error of mean.

**Table 5:** Total percentage areas of different secondary structures in the Amide I region of protein milk dispersions with adjusted κ-carrageenan concentrations subjected to different temperatures (20, 90, or 121 °C) and shear rates (0, 100, or 1000 s^−1^).

κ-Carrageenan Addition (%)	Temp. (°C)	Intramolecular β-Sheets (1615–1637)			Random Coils (1638–1645)			α-Helix (1646–1664)			β-Turns (1665–1681)			Aggregated β-Sheets (1682–1700)		
		0 s^−1^	100 s^−1^	1000 s^−1^	0 s^−1^	100 s^−1^	1000 s^−1^	0 s^−1^	100 s^−1^	1000 s^−1^	0 s^−1^	100 s^−1^	1000 s^−1^	0 s^−1^	100 s^−1^	1000 s^−1^
0	20	50.41 ^A^			6.41 ^H^			31.01 ^A^			8.15 ^I^			3.51 ^J^		
	90	38.89 ^Da^	31.45 ^Bb^	25.49 ^Bc^	5.87 ^Ic^	7.87 ^Eb^	11.05 ^Fa^	22.59 ^Ha^	17.62 ^Eb^	12.87 ^Ec^	24.62 ^Dc^	25.71 ^Bb^	30.41 ^Ba^	5.13 ^Ia^	4.72 ^Fb^	3.33 ^Hc^
	121	33.93 ^Ha^	31.94 ^Bb^	25.14 ^Bc^	8.06 ^Fc^	9.17 ^Db^	12.17 ^Da^	13.02 ^Ja^	12.95 ^Ga^	11.70 ^Fb^	31.39 ^Ac^	33.09 ^Ab^	37.43 ^Aa^	6.82 ^Fa^	5.54 ^Eb^	4.14 ^Gc^
0.01	20	43.88 ^B^			10.47 ^A^			29.20 ^B^			16.45 ^H^			5.84 ^H^		
	90	30.51 ^Ia^	28.53 ^Eb^	23.85 ^Dc^	8.00 ^Gc^	9.73 ^Bb^	11.35 ^Ea^	20.07 ^Ia^	16.25 ^Fb^	10.03 ^Gc^	18.35 ^Ga^	17.49 ^Fb^	16.12 ^Ec^	6.18 ^Gc^	6.74 ^Db^	7.81 ^Fa^
	121	23.74 ^La^	23.73 ^Fa^	19.86 ^Fb^	3.60 ^Jc^	6.19 ^Fb^	11.12 ^Fa^	10.27 ^Ka^	9.27 ^Hb^	7.78 ^Hc^	19.39 ^Fa^	18.91 ^Eb^	17.23 ^Dc^	7.82 ^Cc^	8.18 ^Cb^	9.32 ^Ea^
0.03	20	39.01 ^C^			10.14 ^B^			28.16 ^C^			19.61 ^F^			6.73 ^F^		
	90	35.03 ^Ga^	33.46 ^Ab^	24.08 ^Cc^	8.14 ^Ec^	9.54 ^Cb^	12.29 ^Da^	24.63 ^Fa^	21.04 ^Db^	19.63 ^Dc^	24.63 ^Da^	19.96 ^Db^	17.51 ^Dc^	7.92 ^Cc^	8.92 ^Bb^	10.01 ^Ba^
	121	30.90 ^Ia^	29.46 ^Db^	23.06 ^Ec^	3.49 ^Kc^	9.59 ^Cb^	12.97 ^Ca^	25.08 ^Ea^	24.79 ^Aa^	22.28 ^Ab^	26.67 ^Ca^	24.16 ^Cb^	19.67 ^Cc^	8.17 ^Bc^	8.99 ^Bb^	9.52 ^Da^
0.05	20	37.74 ^E^			10.13 ^B^			26.67 ^D^			18.45 ^G^			7.15 ^E^		
	90	36.02 ^Fa^	30.11 ^Cb^	27.75 ^Ac^	9.25 ^Cc^	10.46 ^Ab^	13.76 ^Ba^	25.69 ^Ea^	23.24 ^Bb^	21.72 ^Bc^	21.03 ^Ea^	18.65 ^Eb^	15.76 ^Fc^	7.58 ^Dc^	8.22 ^Cb^	9.83 ^Ca^
	121	29.61 ^Ja^	21.72 ^Gb^	19.14 ^Fc^	8.25 ^Dc^	9.15 ^Db^	14.78 ^Aa^	23.56 ^Ga^	22.09 ^Cb^	20.34 ^Cc^	28.57 ^Ba^	17.86 ^Fb^	10.73 ^Gc^	8.34 ^Ac^	9.81 ^Ab^	10.74 ^Aa^
SEM *		0.025			0.013			0.045			0.037			0.008		

The means in a column with different superscript upper-case letters and in a row with different superscript lower-case letters differ significantly (*p* < 0.05). * Standard error of the mean.

## Data Availability

Data is contained within the article or supplementary material.

## Supplementary Materials

The following supporting information can be downloaded at: https://www.mdpi.com/article/10.3390/foods12244404/s1. Figure S1: SDS-PAGE analysis of supernatants of MPC dispersions.
