# Supplementary material for: Heat-Induced Changes in κ-Carrageenan-Containing Chocolate-Flavoured Milk Protein Concentrate Suspensions under Controlled Shearing
Source: Foods. 2023 Dec 7;12(24):4404. doi: 10.3390/foods12244404 (PMC10742440; doi:10.3390/foods12244404)

Figure S1: SDS-PAGE analysis of supernatants of MPC dispersions non-reducing (A) and reducing (B) of 0% κ-carrageenan (1st raw), non-reducing (C) and reducing (D) of 0.01% κ-carrageenan (2nd raw) , non-reducing (E) and reducing (F) of 0.03% κ-carrageenan (3rd raw) and non-reducing (G) and reducing (H) of 0.05% κ-carrageenan (4th raw) from left to right. Lane sequence: control (25-0)/90-0/90-100/90-1000/121-0/-121-100/121-1000 from left to right.

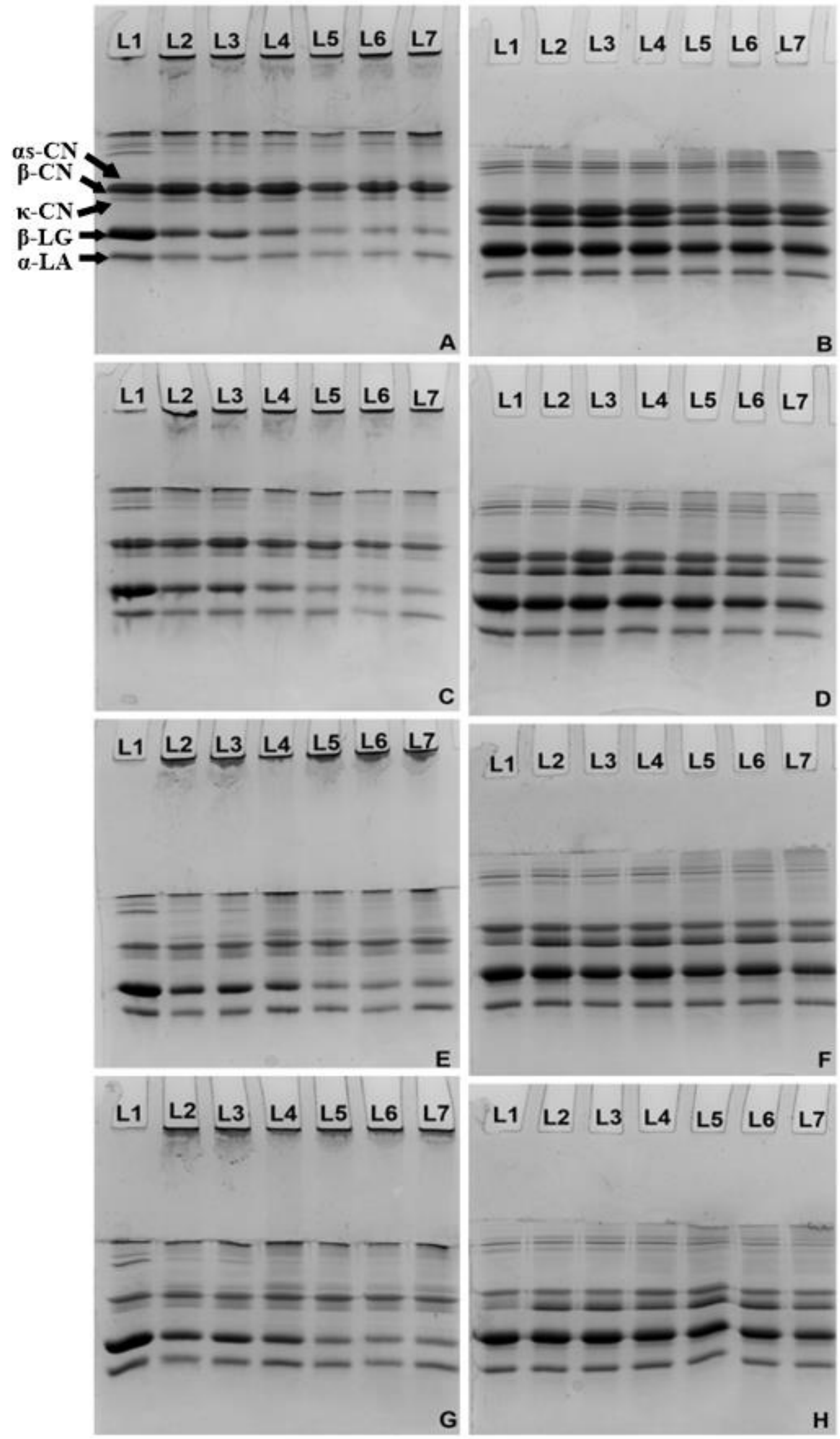

Supplement: Supplementary file 1 [file foods-12-04404-s001.zip › foods-2745794-supplementary.pdf]
